# Dissecting insect cell heterogeneity during influenza VLP production using single-cell transcriptomics

**DOI:** 10.3389/fbioe.2023.1143255

**Published:** 2023-03-06

**Authors:** Marco Silvano, Nikolaus Virgolini, Ricardo Correia, Colin Clarke, Inês A. Isidro, Paula M. Alves, António Roldão

**Affiliations:** ^1^ iBET-Instituto de Biologia Experimental e Tecnológica, Oeiras, Portugal; ^2^ ITQB NOVA, Instituto de Tecnologia Química e Biológica António Xavier, Universidade Nova de Lisboa, Oeiras, Portugal; ^3^ NIBRT-National Institute for Bioprocessing Research and Training, Dublin, Ireland; ^4^ School of Chemical and Bioprocess Engineering, University College Dublin, Dublin, Ireland

**Keywords:** IC-BEVS, influenza VLP, single-cell RNA sequencing, cell heterogeneity, pathway analysis

## Abstract

The insect cell-baculovirus expression vector system (IC-BEVS) has been widely used to produce recombinant protein at high titers, including complex virus-like particles (VPLs). However, cell-to-cell variability upon infection is yet one of the least understood phenomena in virology, and little is known about its impact on production of therapeutic proteins. This study aimed at dissecting insect cell population heterogeneity during production of influenza VLPs in IC-BEVS using single-cell RNA-seq (scRNA-seq). High Five cell population was shown to be heterogeneous even before infection, with cell cycle being one of the factors contributing for this variation. In addition, infected insect cells were clustered according to the timing and level of baculovirus genes expression, with each cluster reporting similar influenza VLPs transgenes (i.e., hemagglutinin and M1) transcript counts. Trajectory analysis enabled to track infection progression throughout pseudotime. Specific pathways such as translation machinery, protein folding, sorting and degradation, endocytosis and energy metabolism were identified as being those which vary the most during insect cell infection and production of Influenza VLPs. Overall, this study lays the ground for the application of scRNA-seq in IC-BEVS processes to isolate relevant biological mechanisms during recombinant protein expression towards its further optimization.

## 1 Introduction

The insect cells (IC) and baculovirus expression vector system (BEVS) constitute an attractive alternative to mammalian cells for manufacturing of heterologous gene products, including recombinant proteins as vaccine candidates and viral vectors for gene therapy (Drugmand et al., 2012). Recent advances in next-generation sequencing technologies have enabled a considerable improvement of our understanding of the IC-BEVS. For example, RNA-seq has been used to assess the transcriptional changes of alphanodavirus-free High Five cells upon infection by *Autographa californica* multiple nucleopolyhedrovirus (*Ac*MNPV) (Chen et al., 2013; Chen et al., 2014), providing a global picture of the AcMNPV transcription regulation throughout the infection cycle. As the knowledge of the IC-BEVS grows, potential engineering targets to increase recombinant protein production are being identified (Silvano et al., 2022).

In recent years, technological advances in areas such as cell isolation methods using microfluidics or microwell devices, preparation of next-generation sequencing libraries from ultra-low quantities of nucleic acids, and innovative labelling strategies for MS-based proteomics have enabled the characterization of DNA, RNA and proteins at single-cell resolution (Lee et al., 2020). Using different single-cell omics profiling strategies as building blocks, we can now build a multi-omics profile of the same cell. These multi-omics methods will play an important role in many diverse fields, and their applications are rapidly expanding, including delineating cellular diversity (Gulati et al., 2020), lineage tracing (Wagner and Klein, 2020), identifying new cell types (Ianevski et al., 2022), and deciphering the regulatory mechanisms between omics (Cao and Gao, 2022). Single-cell analysis has unambiguously demonstrated that cell populations are often heterogeneous (Goldman et al., 2019). This heterogeneity not only applies to different cell types in a tissue (Karlsson et al., 2021) but also to clonal cell population (Stockholm et al., 2007).

Single-cell RNA sequencing (scRNA-seq) has just recently be applied to virus-based processes. The power of scRNA-seq lies in the simultaneous delivery of snapshots of virus and host transcriptomes, and allows to compare host transcriptome between cells with low and high viral loads (Suomalainen and Greber, 2021). The high-resolution dissection of viral and host cell gene expression patterns reveals that the transcriptional responses of individual infected cells can be divergent, as the interplay between underlying cellular heterogeneity and viral population diversity influences the fate of infection (Sun et al., 2020). For example, cell-to-cell variation in viral transcription has been observed during influenza virus infection in mammalian (A549, MDCK and HEK293) cells (Russell et al., 2018).

To date, the understanding of the IC-BEVS transcriptome has been mostly relying on bulk RNA-seq analysis (Nguyen et al., 2013; Chen et al., 2014; Silvano et al., 2022; Virgolini et al., 2022). For instance, we have previously assessed whole transcriptome changes in High Five insect cells during expression of influenza HA-displaying virus-like particles (HA-VLPs) using IC-BEVS, which enabled to identify key biological processes impacted by virus infection (Silvano et al., 2022). Although these studies uncover transcriptional changes in insect cell response to baculovirus infection, they only provide rough models of the host cell response. Understanding IC-BEVS at the single-cell level could elucidate better the mechanisms of viral infection and potentially enable to identify, within a potentially heterogenous cell population and infection process, the characteristics of cells associated with a more efficient progression of infection and production of heterologous proteins.

In this study, we used scRNA-seq for the characterization of the High Five insect cell line during production of influenza HA-VLPs using IC-BEVS. The transcriptomics pipeline here described allowed to study, at a single-cell level, High Five cell population heterogeneity (prior and during infection), host cell response to virus infection, and progression of infection (expression of virus genes and transgenes encoding HA-VLPs).

## 2 Materials and methods

### 2.1 Cell line and culture media

High Five insect cells (Invitrogen) were routinely sub-cultured to .3–.5×10^6^ cell mL^−1^ every 2–3 days when cell concentration reached 2–3×10^6^ cell mL^−1^ in serum-free Insect-XPRESS™ medium (Sartorius) using 125–500 mL shake flasks with a 10% working volume, and maintained at 27°C in a Inova 44R shaking incubator (orbital motion diameter of 2.54 cm—Eppendorf) operating at 100 RPM.

### 2.2 Baculovirus amplification and storage

Recombinant baculoviruses carrying influenza capsid M1 from A/California/06/2009 H1N1 strain and hemagglutinin (HA) from A/Brisbane/59/2007 strain genes were kindly provided by Redbiotec AG (Schlieren, Switzerland). Baculovirus amplification was performed as described elsewhere (Vieira et al., 2005).

### 2.3 Production of HA-displaying VLPs

HA-VLPs production was carried out in a 0.5 L stirred tank bioreactor (BIOSTAT Qplus–Sartorius) as specified elsewhere (Correia et al., 2020). Cells were expanded in 500–2000 mL shake flasks with a 10% working volume as described above. Infection experiments were performed in bioreactor at cell concentration at the time of infection (CCI) of 2×10^6^ cell mL^−1^ and multiplicity of infection (MOI) of 1 pfu.cell^−1^. Medium exchange was performed at the time of infection by centrifugation at 200 g at room temperature for 10 min. Samples were taken every 24 h for the assessment of cell concentration and viability, and detection of M1 and HA proteins; for scRNA-seq, samples were taken before infection, and at 8 and 22 h post-infection (hpi).

### 2.4 Purification of HA-displaying VLPs

Culture bulk from bioreactor run was harvested 3 days post-infection and centrifuged at (first) 4°C, 200 g, for 10 min and (second) four°C, 2000 g, for 20 min. The supernatant was filtered using a .22 µm Stericup (Millipore), and the HA-VLPs were purified using a SartoBind Q capsule (Sartorius Stedim Biotech) according to manufacturer’s instructions. Purified material was formulated in 50 mM HEPES, 300 mM NaCl, 15% (w/v) trehalose, pH 7.4, and stored at −80 or four°C.

### 2.5 Analytics

#### 2.5.1 Cell concentration and viability

Cell concentration was determined in a Fuchs-Rosenthal hemocytometer chamber (Brand) and cell viability assessed by trypan blue exclusion method (J R Tennant, 1964).

#### 2.5.2 Baculovirus titration

Baculovirus titers were determined using the MTT assay as described elsewhere (Mena et al., 2003; Roldão et al., 2009).

#### 2.5.3 Western blot

Identification and relative quantification of M1 and HA in culture supernatant were performed as reported elsewhere (Correia et al., 2020).

#### 2.5.4 Transmission electron microscopy

Negative staining transmission electron microscopy was used to assess the conformation and size of HA-VLPs. Briefly, 5 μl of purified VLP sample was fixed for 2 min in a copper grid coated with Formvar-carbon (Electron Microscopy Sciences, Hatfield). Grids were washed with H2O and then stained with 2% (v/v) uranyl acetate for 5 min and left to air dry. Samples were then observed in a Hitachi H-7650 Transmission Electron Microscope (JEOL, United States).

### 2.6 Single-cell RNA sequencing

For single-cell gene expression profiling, ≈6000 cells (at 0 hpi and 8 hpi) or ≈8000 cells (at 22 hpi) were loaded into a BD Rhapsody cartridge (BD Biosciences) and libraries were generated according to BD Rhapsody™ System mRNA Whole Transcriptome Analysis (WTA). Upon confirming the quality of the resulting libraries using a Bioanalyser, the quantity of each library was determined using Qubit. ScRNA-seq libraries were sequenced using an Illumina NovaSeq (Illumina) configured to yield 150 bp paired end reads.

### 2.7 Single-cell RNA data analysis

#### 2.7.1 Generation of a UMI count matrix

The cellular barcodes were pre-processed and demultiplexed by the BD Rhapsody WTA bioinformatic workflow (BD Biosciences) on the Seven Bridges Genomics (SBG) cloud platform using default parameters, as reported elsewhere (Tzani et al., 2021). STAR indexes were generated from the *Trichoplusia ni* (*Tnl*) reference genome (RefSeq assembly accession: GCF_003590095.1) and from *AcMNPV* (RefSeq assembly accession: GCF_000838,485.1, ViralProj14023) (Dobin et al., 2013). Specifically, a hybrid reference genome was used for RNA-seq read mapping using transgenes (M1 and HA) sequences and mtDNA sequence of the *Tnl* reference genome (GenBank accession No. MK714850.1).

#### 2.7.2 Filtering the UMI count matrix

The cell/gene matrices output from the SBG pipeline were imported into the R-4.2.1 Statistical Software Environment and merged to form a single matrix for further analysis. The proportion of unique molecular indexes (UMIs) originating from mtDNA was also determined for each cellular barcode, and cells with >5% mitochondrial UMIs counts were considered of low-quality and thus removed from further analysis.

#### 2.7.3 UMAP and pseudotime analysis

Seurat v4 was used to apply a graph-based clustering approach (Hao et al., 2021). These methods embed cells in a graph structure with edges drawn between cells with similar feature expression patterns, and then attempt to partition this graph into highly interconnected “communities” (Xu and Su, 2015). To evaluate cell heterogeneity, data sets (0, 8, and 22 hpi) were merged prior to global scaling normalization method. Normalized and merged samples were scaled and variations caused by different total UMIs per cell were regressed out. The most variable features were considered for principal component analysis, and 20 principal components were used to perform cluster analysis. The Uniform Manifold Approximation and Projection (UMAP) technique was used to run non-linear dimensional reduction and to visualize and explore the datasets (McInnes et al., 2018). Monocle three was run to conduct trajectory analysis (Trapnell et al., 2014) and its function graph_test was used to identify genes that change as function of pseudotime. Genes with an average expression change of **≥** .5 and *p*-value <.05 were considered significant.

#### 2.7.4 Cell cycle correction

Cell cycle scoring function in Seurat v4 was used to determine the likelihood of cells being in either S or G2/M phase, based on reference genes known to play a role in distinct phases of the cell cycle. To conduct this procedure, we mapped the mouse gene list to the Tnl genome to carry out the classification and draw a list of Tnl cell-cycle genes. The resulting scores for S, G2/M and G1 phases were used to regress out the effect of cell cycle in downstream analysis.

#### 2.7.5 Functional annotation and enrichment analysis

For gene annotation, the amino acid sequence of protein-coding genes was used as a query. Blastp search was applied in the NCBI nr protein database using Blast2GO OmicsBox (Götz et al., 2008). No taxonomy filter was applied, and the E-value cutoff was set to 1 × 10^−3^. The over-representation of pathways within gene lists found to have a statistically significant association with pseudotime were identified using the Fisher’s exact test. Pathway terms with a False Discovery Rate of <.05 were considered significant (Benjaminit and Hochberg, 1995).

## 3 Results

### 3.1 Production of influenza HA-VLPs

**FIGURE 1:**
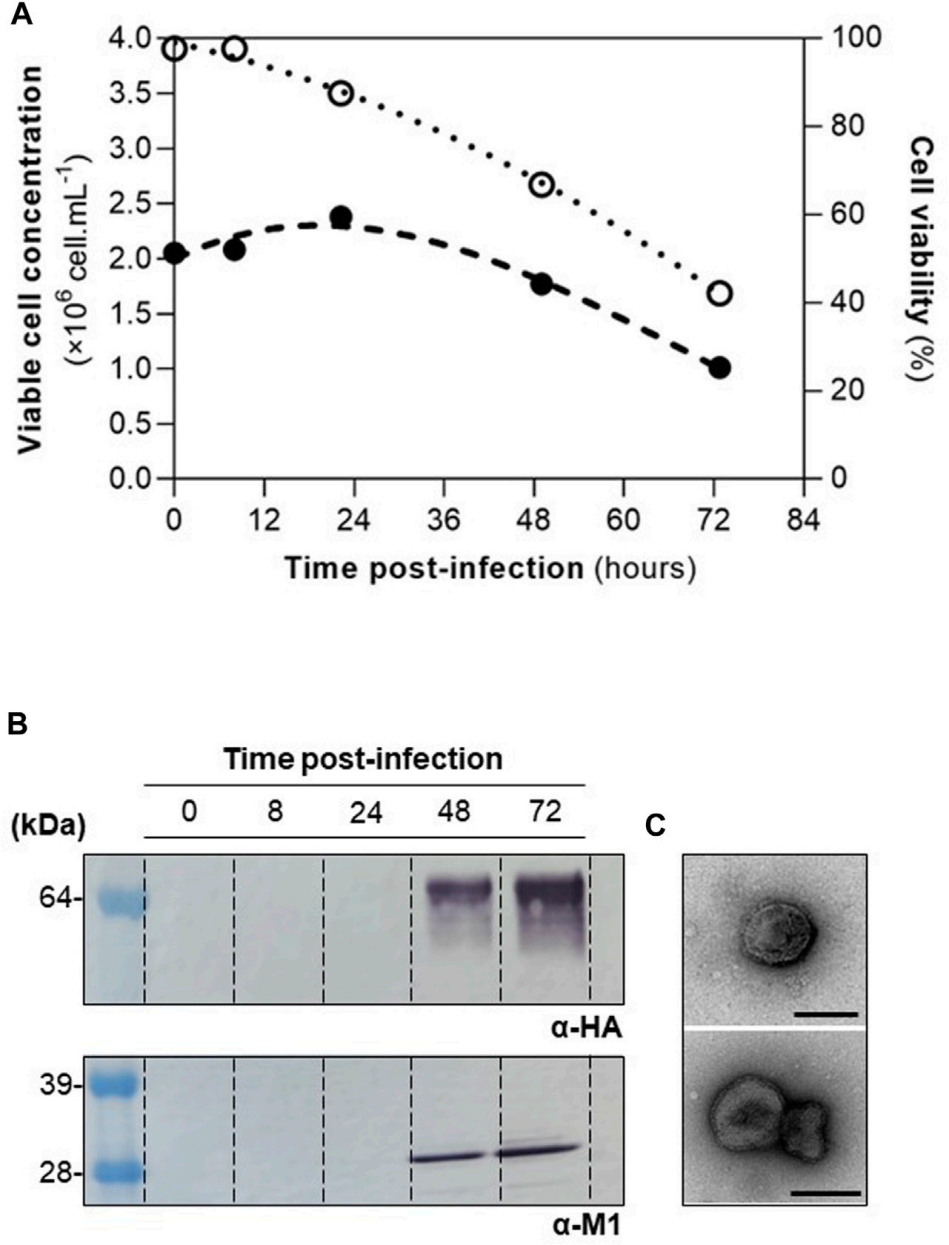
Production of influenza HA-VLPs. **(A)** Cell growth kinetics upon infection. Viable cell concentration and cell viability in full and empty circles, respectively. **(B)** Identification of HA and M1 proteins by Western blot. **(C)** TEM of purified HA-VLPs; scale bar represents 100 nm.

High Five cells were infected at CCI of 2×10^6^ cell mL^−1^ with a MOI of 1 pfu.cell^−1^, and infection kinetics were assessed throughout (Figure 1A). M1 and HA proteins were identified by Western blot (Figure 1B), and particles resembling influenza HA-VLPs, both in size and morphology, detected by TEM (Figure 1C). These results are in line with those previously reported (Silvano et al., 2022) and demonstrate that HA-VLPs were successfully produced.

### 3.2 Single-cell RNA-seq data processing and quality control

For scRNA-seq, ∼6,000–8,000 cells were collected at each time point (>90% cell viability). After sequencing, an average of 391 million 150 bp-reads were acquired for each sample. Following the completion of this initial pre-processing stage, approx. 4% of the sequenced RNA-seq reads were removed from further analysis due to insufficient read length, low base quality and/or high single nucleotide frequency; an average of ≈377 million reads per time point remained valid for further analysis (Supplementary Table S1). From the reads that passed quality control, 87% of the reads were successfully assigned to cell barcodes following demultiplexing. Mapping to the reference genome resulted in a unique alignment rate of ∼71%. Upon collapsing to UMIs and application of the RSEC algorithm, between 4,496 and 5,408 unique cell barcodes were identified for the three samples taken throughout the process. The mean number of reads and mRNA molecules detected per cell in this experiment were 41,166 and 28,787, respectively, with an average of ∼3,372 genes detected in each cell (Supplementary Table S1).

**FIGURE 2:**
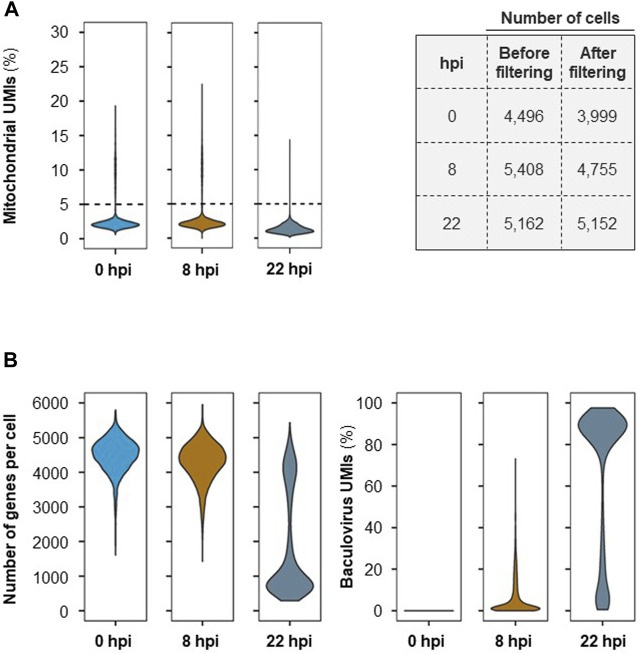
Single-cell RNA-seq quality control. **(A)** Mitochondrial UMIs per cell (on the left) and number of cells before and after filtering per UMIs originating from mitochondrial genes (>5%) (on the right). **(B)** Number of genes identified per cell and percentage (%) of cells containing UMIs originating from baculovirus genes at each time point after cell filtering.

To ensure that only high-quality genes were retained for further analysis, the UMI count matrix was filtered to remove data that might have originated from non-viable cells. An average of 8% of cells contained >5% of detected UMIs originating from mitochondrial genes and thus were eliminated from further analysis (Figure 2A). The number of genes per cell and baculovirus UMIs (Figure 2B) as well as the amount of total UMIs per cell (Supplementary Figure S1) were assessed; while at 0 and 8 hpi the average number of genes identified per cell is 4000–5000, this number decreases significantly at later stages of infection (i.e., 22 hpi) concomitantly with an increase in the percentage of baculovirus UMIs.

### 3.3 High Five insect cells heterogeneity

**FIGURE 3:**
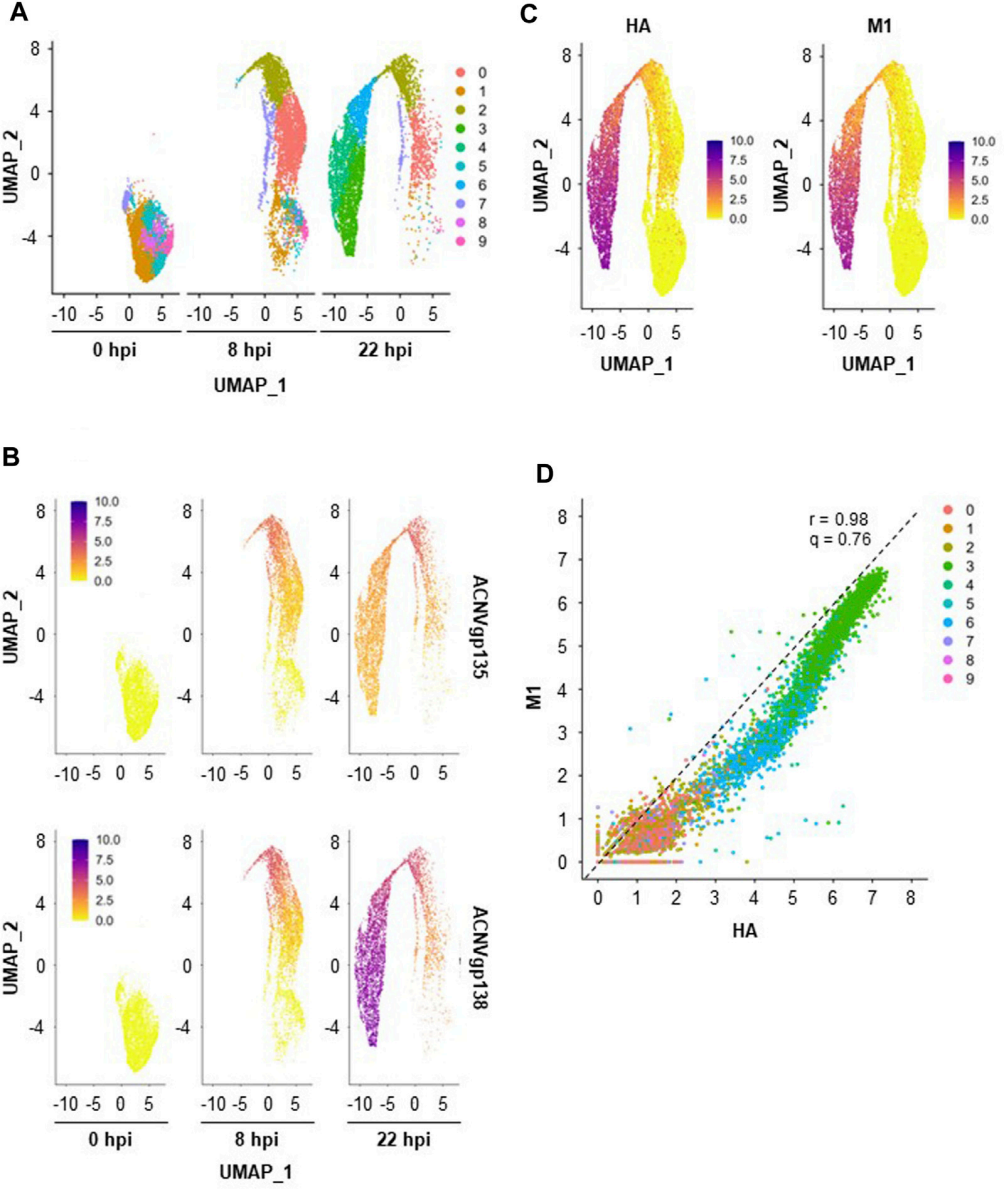
Insect cells clustering and transgenes expression. **(A)** Merged scRNA-seq dataset obtained with UMAP. **(B)** Relative expression of early (*ACNVgp135*) and late (*ACNVgp138*) baculovirus genes. **(C)** Relative expression of transgenes HA and M1. A color gradient scale was used to show the relative gene expression per cell. **(D)** Relative HA and M expression per cell, with dashed line representing the best linear fit to the data (r—Pearson’s correlation coefficient, q–angular coefficient). A color code was used to identify each cell cluster.

To identify cell populations existing across the three single-cell datasets (0, 8, and 22 hpi), merged analysis was performed using Seurat v4. A total of 10 clusters were drawn from this analysis (Figure 3A), with a noticeable cluster re-organization being observed throughout infection progression. Importantly, five of those clusters were already present before infection (0 hpi) suggesting that High Five cells population was heterogeneous. Cell cycle is known to contribute to heterogeneity in scRNA-seq datasets (McDavid et al., 2016); to ascertain this in our study, cell cycle covariate was estimated using the Cell Cycle Scoring method in the Seurat package (Tirosh et al., 2016). While heterogeneity is clear at 0 hpi, with cells being associated to different cell cycle stages in a proportion of ≈1:7:5 (S:G2M:G1), at 22 hpi most cells have been identified as being in G1 phase (74%) (Supplementary Figure S2A,B). However, at this later timepoint, cell cycle association seems to be misassigned as exemplified by the overall low expression of the G1 cell cycle-related genes *ccnd3*, *ccne1,* and *cdk6* in Supplementary Figure S2C. This could be a consequence of overexpression of baculovirus UMIs (hence lower expression of host cell genes), impairing correct cell cycle identification. Thus, the cell cycle regression was not further applied for the merged dataset.

Cluster re-organization throughout infection seems to be correlated with the expression of baculovirus genes as infection progresses (Figure 3B); for example, expression of an early baculovirus gene (*ACNVgp135*) was higher in clusters denoting a transitory stage (i.e., cluster #2) whereas a late baculovirus gene (*ACNVgp138*) was more expressed in clusters (e.g., clusters #3, #4, and #6) furthest from those identified at 0 hpi. As seen for *ACNVgp138*, expression of transgenes M1 and HA, both under the regulation of the late expression promoter polyhedrin, was mainly identified in clusters #3, #4, and #6 (Figure 3C), with similar expression levels of HA and M1 genes being observed regardless of the cell cluster (r = .98, q = .76, Figure 3D).

### 3.4 Pseudo-temporal ordering of cells after infection

**FIGURE 4:**
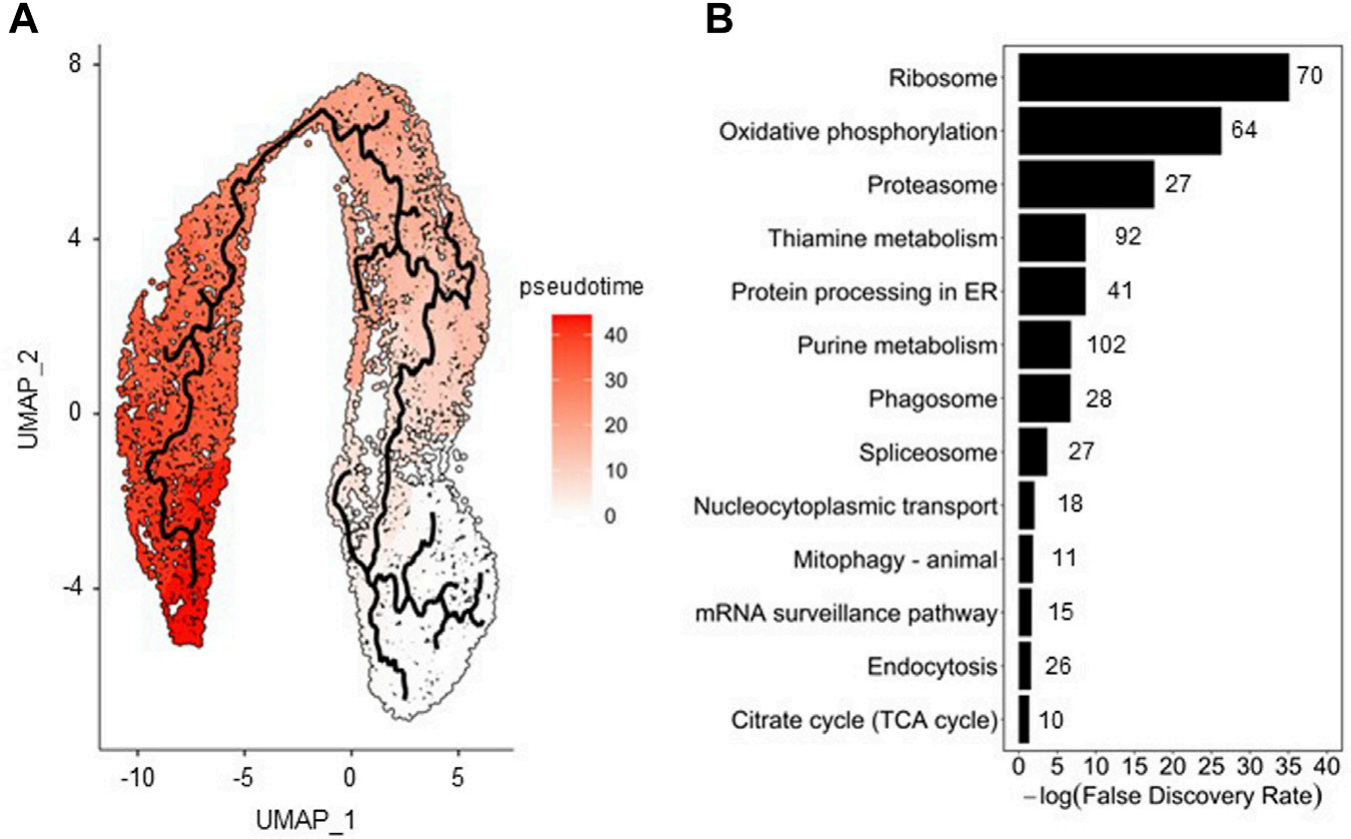
Pseudotime analysis of insect cells along infection. **(A)** Trajectory analysis on merged cell dataset (0, 8, and 22 hpi); a color gradient scale was used to visualize the pseudotime. **(B)** Pathway enrichment analysis performed with genes identified as the most changing in function of pseudotime using the Fisher’s exact test; the number of genes involved and found in the enrichment analysis are shown next to the bars.

To assess cell population evolution during infection, pseudo-temporal ordering (i.e., trajectory analysis) was applied. To perform this analysis, 13,906 cells from the merged Seurat analysis were used as input of Monocle 3. The cell cluster with the lowest percentage of HA (cluster #5, see Figure 3A) was selected as root state of the trajectory since it was the one most closely resembling the non-infected cell population; the pseudotime variable was then ordered accordingly (Figure 4A).

Transcriptomic changes characterizing the progression of cells along HA production were assessed through identification of genes correlated with pseudotime. Overall, 921 host cell genes were found to be significantly (q value <.05) associated with pseudotime. Pathway enrichment analysis allowed to identify biological processes varying most along infection, of which those associated to translation machinery, energy metabolism, protein folding and endocytosis (Figure 4B) are some examples, in good agreement to what we have previously found in bulk RNA sequencing analysis (Silvano et al., 2022; Virgolini et al., 2022). The relative expression of a selected number of genes involved in these pathways is presented in Supplementary Figure S3, illustrating the significant transcriptional changes in cells upon infection.

## 4 Discussion

In this work, we used scRNA-seq sequencing to analyze, at the single-cell level, alterations in the transcriptome of a High Five insect cell line infected with baculovirus during the production of influenza HA-VLPs.

Stable and high foreign gene expression levels are important criteria during the development of producer cell lines for pharmaceutical applications (Tripathi and Shrivastava, 2019), and understanding the mechanisms behind gene expression variation of genetically identical cells is the first step of this process. Cell population heterogeneity can influence parameters such as cell growth rate, genetic stability and productivity (Schmitz et al., 2019), and thus tracking it is key to avoid process failure and guarantee reproducibility (Olsson et al., 2022). Most studies on this topic focus on mammalian cells (Lewis et al., 2016; Samoudi et al., 2021), with none to date exploring insect cells. In our study, we could observe heterogeneity in High Five cell population before infection (as demonstrated by the identification of five cell clusters), largely resulting from the phase of the cell cycle that the cells are in, which was further amplified upon infection (6 more cell clusters were identified) as consequence of viral DNA replication and gene expression (Du and Thiem, 1997) and cell cycle arrest (Braunagel et al., 1998).

Baculovirus genes are known to be transcribed temporally, a process highly regulated by infection-derived mechanisms and mediated by host and viral protein expression (Nguyen et al., 2013). The timing and level of baculovirus gene expression were herein identified as the main factors driving clustering of infected insect cells. Trajectory analysis allowed us to confirm this, in which a clear path along pseudotime is observed although cells separate and order across multiple branches spanning the transcriptomic space.

Biological mechanisms associated with baculovirus infection and transgenes (in our case those coding for influenza HA-VLPs) expression can be identified by correlating changes in gene(s) expression to progression of cells along the infection trajectory. Through pathway enrichment analysis, we found the endocytosis pathway as being one of the most significantly enriched biological processes during infection, which derives from viruses exploiting cellular structures towards endocytosis-mediated viral nucleocapsid transport to the nucleus (Monteiro et al., 2012). In addition, entry of baculovirus is found dependent on clathrin-mediated endocytosis (Long et al., 2006), which was herein corroborated by the upregulation of clathrin *cltc* and actin-related *arpc5* and *capza1* proteins at early infection stages.

Among the cellular defense responses to environmental and pharmacological stresses, the activation of heat shock response (HSR) is one of the most important. It leads to rapid and robust expression of members of the chaperone family of heat shock proteins (HSPs) in order to protect the cell from proteotoxic stresses and to maintain protein homeostasis (Fujimoto and Nakai, 2010). Interestingly, viruses can exploit HSR as an infection strategy, making use of HSPs such as HSP70 and HSP90 for regulation of viral gene expression and capsid assembly/disassembly (Mayer and Bukau, 2005; Xiao et al., 2010; Nagy et al., 2011). Our data corroborates this, with the expression of *hsp70* found significantly upregulated early in infection. In addition, the proteasome pathway was found enriched, in agreement with the reported evidence of close collaboration between HSPs and ubiquitin-proteasome system during the baculovirus replicative cycle (Katsuma et al., 2011).

Baculovirus infection induces an important metabolic burden on insect cells, enhancing the fluxes through the major catabolic pathways including the tricarboxylic acid cycle (TCA) (Bernal et al., 2009). Within the TCA cycle, the citrate synthase *cs* gene is involved in aerobic energy production and metabolic interconversions in mitochondria (Holloszy et al., 1970); in our analysis, the expression of *cs* was found significantly increased at the onset of infection, suggesting that this gene plays a key role as a first-line response to infection.

Overall, the enrichment analysis allowed to identify several pathways (e.g., ribosome, spliceosome, oxidative phosphorylation) that were common to those previously identified in our bulk RNA sequencing study (Silvano et al., 2022), demonstrating the robustness and replicability of the data. Importantly, single-cell RNA sequencing allowed to evaluate single cells at different states of infection within the same sample and capture the transcriptional changes associated with the infection process (not possible with the bulk RNA sequencing approach), thus elevating the importance of single-cell omics analysis in the IC-BEVS system.

## 5 Conclusion

Single-cell transcriptomics enabled us to study host cell and baculovirus gene expression patterns at a resolution previously unobtainable in a bulk approach, allowing to isolate traces of different stages of infection progression. Such understanding can be further applied through genetic engineering approaches for overexpression/knock-out of specific genes, an approach that opens possibilities such as developing cell lines specialized for either virus replication or foreign protein expression, establish inducible systems, and even stimulate infection synchronization across all cells in culture towards a more controlled, homogeneous production process. Notwithstanding, using scRNA-seq to study additional IC-BEVS processes (i.e., comprising different products of interest, infection conditions, among others) is crucial for a more broader understanding of the transcriptome footprints of this expression system.

## Data Availability

The datasets presented in this study can be found in online repositories. The names of the repository/repositories and accession number(s) can be found below: https://www.ncbi.nlm.nih.gov/, PRJNA911494. The sensitive nature of some of the reagents used in this study (e.g., cell lines, plasmids, baculoviruses, and antibodies) means that they are only readily available internally to the author’s institutions staff for the R&D purposes. For external researchers, approval of reagents request may be obtained *via* e-mail addressed to the corresponding author.

## Supplementary material

Supplementary Figure S1 Number of total UMIs per cell at each time point after cell filtering.

Supplementary Figure S2 Cell cycle association to non-infected and infected insect cells. **(A)** Cell cycle score assignment for merged scRNA-seq dataset. **(B)** Proportion of cells assigned to each cell cycle phase. Pink, green and blue are the colors assigned to cells in G2M, S, G1, respectively. **(C)** Relative expression of genes involved in G1 phase: Cyclin D3 (*ccdn3*), Cyclin D1 (*ccne1*), and Cyclin-dependent kinase 6 (*cdk6*).

Supplementary Figure S3 Relative expression of genes associated to pathways found enriched in pseudotime analysis. Cltc, arpc5, and capza1 are genes involved in the endocytosis pathway; hsp70, psmb1, and cs are genes involved in the protein processing in ER, proteasome and citrate cycle (TCA cycle), respectively. A color gradient scale was used to show the relative gene expression per cell.
