# Supplementary figures and images for: Dissecting insect cell heterogeneity during influenza VLP production using single-cell transcriptomics
Source: Front Bioeng Biotechnol. 2023 Mar 6;11:1143255. doi: 10.3389/fbioe.2023.1143255 (PMC10025388; doi:10.3389/fbioe.2023.1143255)

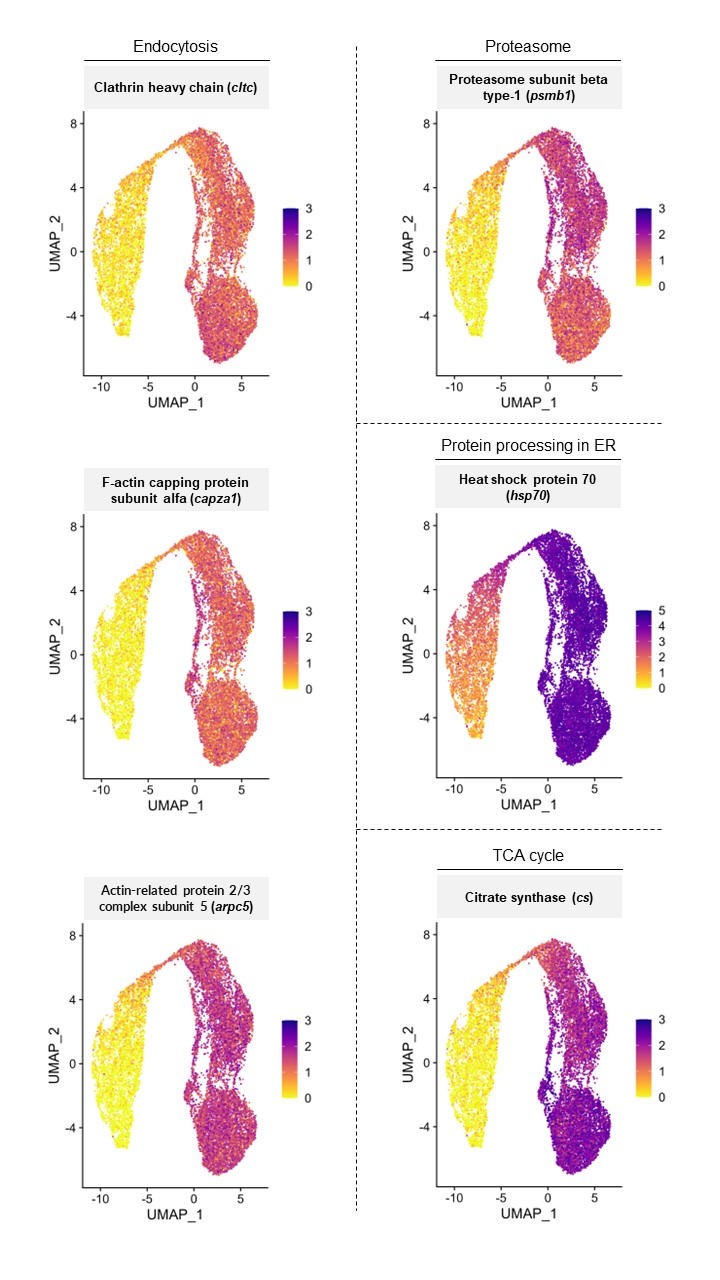

Supplement: Supplementary file 1 [file Image3.JPEG]

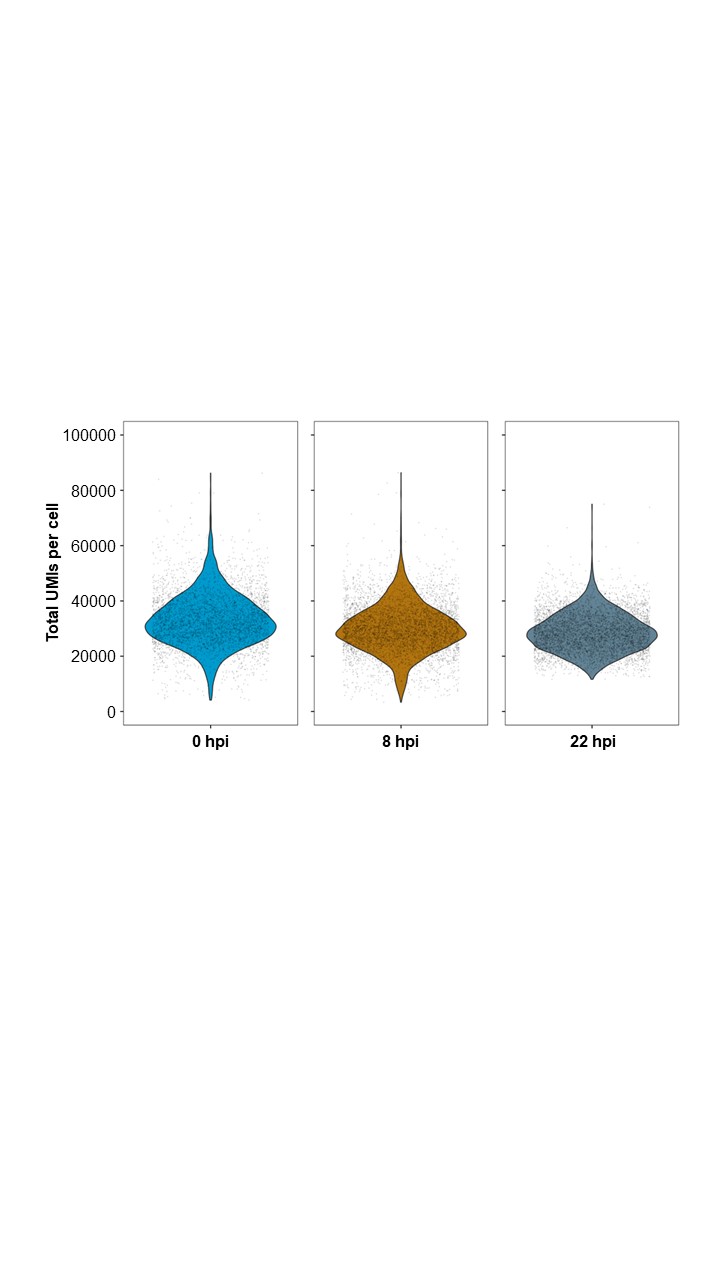

Supplement: Supplementary file 3 [file Image1.JPEG]

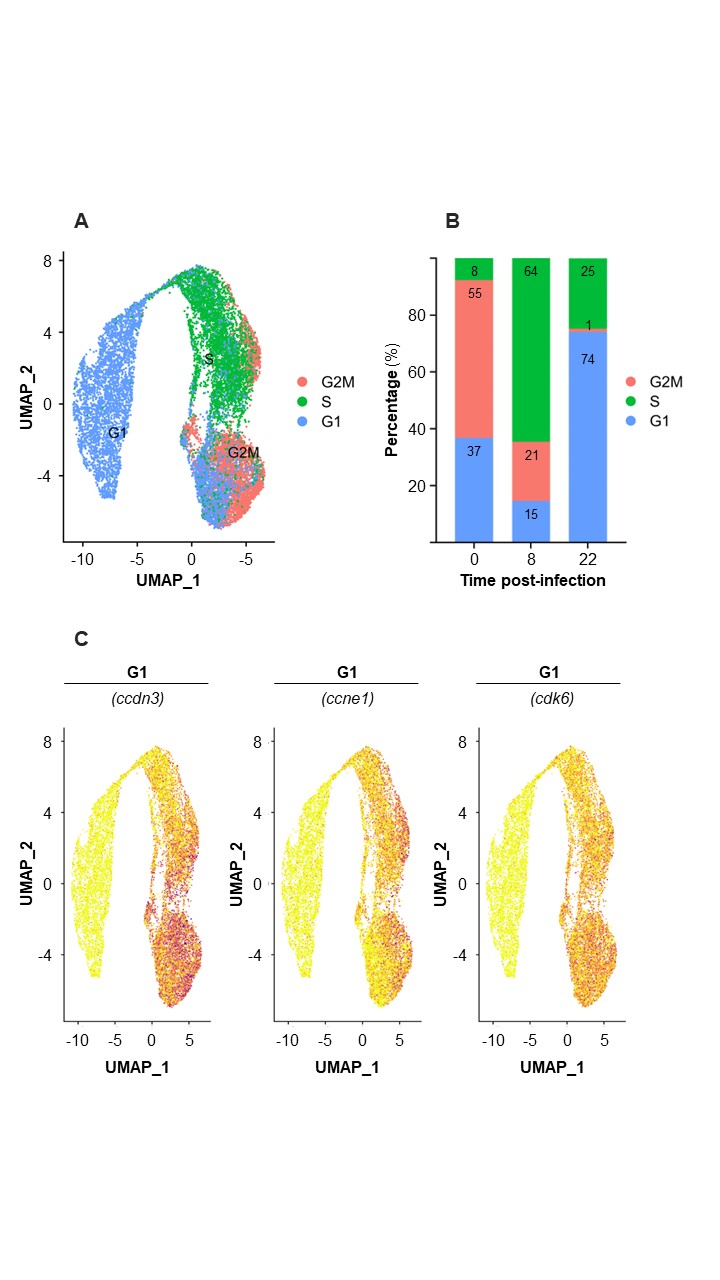

Supplement: Supplementary file 4 [file Image2.JPEG]
