# Supplementary material for: Dissecting insect cell heterogeneity during influenza VLP production using single-cell transcriptomics
Source: Front Bioeng Biotechnol. 2023 Mar 6;11:1143255. doi: 10.3389/fbioe.2023.1143255 (PMC10025388; doi:10.3389/fbioe.2023.1143255)
Supplement: Supplementary file 2 [file Table1.docx]

**Supplementary**

| **Metric** | **Category** | **0 hp** | **8 hpi** | **22 hpi** |
| --- | --- | --- | --- | --- |
| **Total Reads in FASTQ** | Sequencing Quality | 367962931 | 365633934 | 438009614 |
| **Pct_Reads_Too_Short** |  | 0 | 0 | 0 |
| **Pct_Reads_Low_Base_Quality** |  | 0.19 | 0.15 | 0.22 |
| **Pct_Reads_High_SNF** |  | 3.27 | 3.88 | 2.89 |
| **Pct_Reads_Filtered_Out** |  | 3.45 | 4.02 | 3.1 |
| **Total_Reads_After_Quality_Filtering** |  | 355254767 | 350931123 | 424421367 |
| **Total_Filtered_Reads** | Library Quality | 355254767 | 350931123 | 424421367 |
| **Pct_Q30_Bases_in_Filtered_R2** |  | 87.7 | 88.24 | 87.75 |
| **Pct_Assigned_to_Cell_Labels** |  | 85.18 | 86.16 | 86.93 |
| **Pct_Cellular_Reads_Aligned_Uniquely_to_Annotated_Transcriptome** |  | 59.4 | 62.0 | 69.28 |
| **Pct_Cellular_Reads_Aligned_Uniquely_to_Other_Genomic_Regions** |  | 9.01 | 8.44 | 5.96 |
| **Pct_Cellular_Reads_Aligned_Not_Unique** |  | 9.88 | 9.66 | 4.05 |
| **Pct_Cellular_Reads_Unaligned** |  | 1.22 | 1.19 | 3.57 |
| **Aligned_Reads_By_Type** | Reads and Molecules | 211015826 | 217590582 | 289780052 |
| **Total_Raw_Molecules** |  | 152663919 | 167853173 | 215652221 |
| **Total_RSEC_Molecules** |  | 149874976 | 165304754 | 192699547 |
| **Mean_Raw_Sequencing_Depth** |  | 1.38 | 1.3 | 1.34 |
| **Mean_RSEC_Sequencing_Depth** |  | 1.41 | 1.32 | 1.5 |
| **Sequencing_Saturation** |  | 49.22 | 41.8 | 51.38 |
| **Putative_Cell_Count** | Cells RSEC | 4496 | 5408 | 5162 |
| **Pct_Reads_from_Putative_Cells** |  | 91.46 | 89.64 | 79.28 |
| **Mean_Reads_per_Cell** |  | 42925.1 | 36067.14 | 44505.76 |
| **Mean_Molecules_per_Cell** |  | 30425.64 | 27346.1 | 28591.16 |
| **Median_Molecules_per_Cell** |  | 31069.5 | 28145.5 | 28130 |
| **Mean_Targets_per_Cell** |  | 4251.6 | 4053.64 | 1812.21 |
| **Median_Targets_per_Cell** |  | 4472 | 4266 | 966.5 |
| **Total_Targets_Detected** |  | 15095 | 15078 | 14722 |

**Table S1. Single-cell RNA-seq metrics.** The outputs from the Seven Bridges Genomics bioinformatics pipeline are shown including the read-processing steps and deconvolution of cell barcodes for each sample.
